# The Clinical Cases of Geleophysic Dysplasia: One Gene, Different Phenotypes

**DOI:** 10.1155/2018/8212417

**Published:** 2018-07-03

**Authors:** Evgenia Globa, Nataliya Zelinska, Andrew Dauber

**Affiliations:** ^1^Ukrainian Research Center of Endocrine Surgery, Pediatric Endocrinology Department, Kyiv, Ukraine; ^2^Division of Endocrinology, Cincinnati Center for Growth Disorders, Cincinnati Children's Hospital Medical Center, USA

## Abstract

**Background:**

Geleophysic dysplasia is a rare multisystem disorder that principally affects the bones, joints, heart, and skin. This condition is inherited either in an autosomal dominant pattern due to* FBN1* mutations or in an autosomal recessive pattern due to* ADAMTSL2 *mutations. Two patients with unaffected parents from unrelated families presented to their endocrinologist with severe short stature, resistant to growth hormone treatment. Routine endocrine tests did not reveal an underlying etiology. Exome sequencing was performed in each family. Our two patients, harboring* de novo* heterozygous* FBN1* mutations p.Tyr1696Asp and p.Cys1748Ser, had common clinical symptoms such as severe short stature, characteristic facial features, short hands and feet, and limitation of joint movement. However, one patient had severe cardiac involvement whereas the other patient had tracheal stenosis requiring tracheostomy placement.

**Conclusions:**

Patients with severe dwarfism, skeletal anomalies, and other specific syndromic features (e.g., tracheal stenosis and cardiac valvulopathy) should undergo genetic testing to exclude acromelic dysplasia syndromes.

## 1. Introduction

Geleophysic dysplasia (GD) (MIM #231050, 617809, and 614185), acromicric dysplasia (AD) (MIM #102370), and Weill-Marchesani syndrome (WMS) (MIM #614819, 608328, 613195, and 277600) are rare disorders with overlapping characteristics including short stature, short hands and feet, progressive joint limitations and contractures, skin thickening, distinctive facial features, and abnormal skeletal morphology [1–7]. AD usually has a less severe outcome due to the absence of progressive cardiac valvular thickening, whereas a distinctive feature in WMS patients is an abnormality of the lens of the eye [1].

GD is characterized by short stature, prominent abnormalities in hands and feet, bones, and joints, and progressive cardiac, respiratory, and lung pathology with a characteristic facial appearance (described as* gelios* = happy, and* physis* = nature) [1–4]. The three genes known to be associated with geleophysic dysplasia are* ADAMTSL2* (geleophysic dysplasia 1),* FBN1* (geleophysic dysplasia 2), and the recently discovered latent transforming growth factor beta (TGF*β*)-binding protein-3 (*LTBP3*) [5–7].* FBN1* encodes fibrillin-1 and* ADAMTSL2* (a disintegrin and metalloproteinase with thrombospondin repeats-like 2) encodes a glycoprotein of unknown function. All* FBN1* pathogenic variants identified to date in GD are clustered in the same region encoding the TGF*β*-binding protein-like domain 5 (TB5) domain of* FBN1* [3, 6].

Mutations in* FBN1* are also associated with a large spectrum of diseases including Marfan syndrome, Weill-Marchesani syndrome, Stiff skin syndrome, MASS syndrome, and Marfan lipodystrophy syndrome [8–10]. The mechanism by which changes in this gene contribute to both tall and short stature is still unclear [3].

Transmission of GD is variable and corresponds to an autosomal recessive model in the cases with* ADAMTSL2* gene mutations and an autosomal dominant model in the cases with* FBN1* and* LTBP3 *mutations [1, 7]. The true prevalence of GD in the world is unknown; however approximately 55 affected individuals have been reported in 2009 [1] giving a prevalence <1/1 000 000. Symptoms of GD can vary widely from person to person and thus the diagnosis of this rare condition can be quite complicated. Many patients may remain un- or misdiagnosed.

In the present study, we report two patients from unrelated families who had highly variable clinical presentations of GD but their common feature was severe short stature with resistance to growth hormone (GH) treatment. Nevertheless, exome sequencing identified the presence of* de novo* heterozygous* FBN1* variants in both patients. This study was approved by the Institutional Review Board at Cincinnati Children's Hospital Medical Center (Protocol #2014-5919). Written informed consent was obtained from both patients' parents.

## 2. Case Presentation

**Figure 1:**
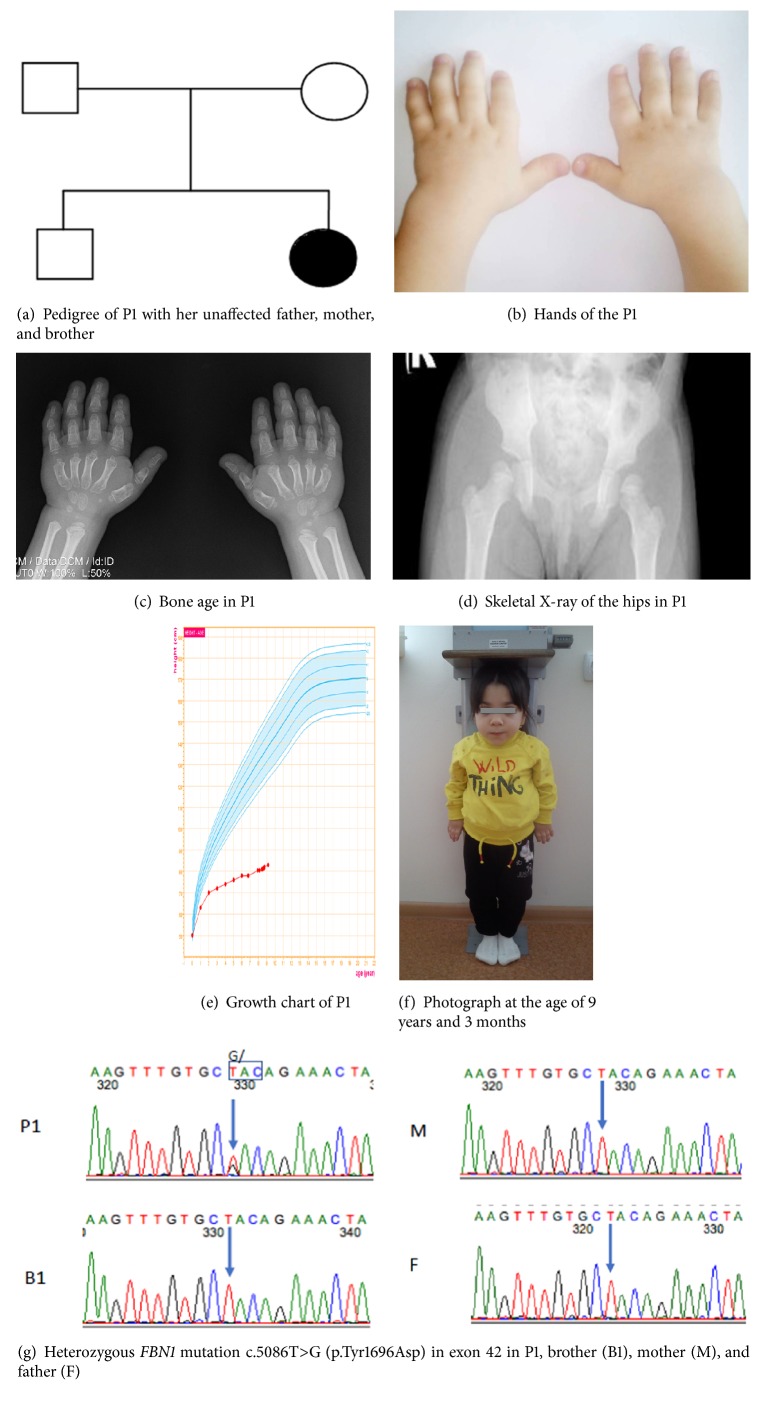


Patient 1 (P1) is a Ukrainian girl who was born as the second child of nonconsanguineous white parents following an uneventful pregnancy and spontaneous term delivery. She had been born at a gestational age of 39 weeks with a normal birth weight (3100 g, 15^th^-50^th^ percentile) and birth length (50 сm, 50^th^-85^th^ percentile). Progressive postnatal growth delay developed since 1 year of age. Familial stature was well within the normal range with a maternal height of 154 cm (-1.3 SD), paternal height of 175 cm (+0.04 SD), and a brother's final height of 169 cm (-0.8 SD) (Figure 1(a)). The patient was first examined by an endocrinologist at the age of 2.5 years. Her height was 72 cm (-4.5 SD) and weight was 7.9 kg (<5^th^ percentile). Physical examination revealed additional dysmorphic features and other physical abnormalities including a broad nasal bridge, a bulbous nose, elongation of the eye lashes, contractures of the elbow joints and wrists, and small hands and fingers (Figure 1(b)). Routine biochemical analysis demonstrated normal hematology, chemistry, and thyroid hormone function. IGF-1 was 51.7 ng/ml (between the 10th and 50th percentile).

Further investigation was done at the age of 6 years and revealed a normal clonidine-stimulated GH peak of 25 ng/ml. Baseline IGF-1 was 74.4 ng/ml (between the 10th and 50th percentile). IGF-1 generation test showed no response to GH stimulation (after three days of GH administration at a dose 0.03 mg/kg/day IGF-1 remained low at 55.2 ng/ml). Karyotype was that of a normal female (46 XX). Her bone age at her calendar age of 6 years was 2 years (as assessed by the Greulich and Pyle method), and cone-shaped epiphyses and Madelung deformity were noted (Figures 1(b) and 1(c)). Skeletal X-ray of the lower extremities showed lateral positioning of the femoral heads and varus deformity of the knee joints (Figure 1(d)). A brain MRI revealed a hypoplastic pituitary and sella turcica. She was prescribed empiric treatment with recombinant GH (rGH) at a dose 0.03 mg/kg/day which was ineffective. Over a period of 4 months, the child grew by only 0.8 cm. Thereafter, rGH therapy was discontinued. Further observation showed progressive growth retardation (Figures 1(e) and 1(f)). At 8 years of age, her height was 80.5 cm (-8 SD) and weight was 11 kg (<5^th^ percentile). At 7 years of age, echocardiography showed minimal aortic, mitral, and pulmonary stenosis. However, two years later, a repeat echocardiogram showed worsening cardiac disease with progression of the mild aortic, mitral, and pulmonary stenosis and new findings of pulmonary hypertension and left ventricular hypertrophy. The patient also suffers from carpal tunnel syndrome which was confirmed using electromyography.

Comprehensive genetic testing was done at 8 years of age. A chromosomal microarray (Illumina CytoSNP-850v1.1) was performed and excluded any pathogenic copy number variants. Subsequently, whole exome sequencing was performed at Cincinnati Children's Hospital on the patient and her parents using previously described methods [11]. As neither parent was affected, both recessive and* de novo* dominant inheritance models were investigated. The patient was found to have a* de novo* heterozygous mutation in* FBN1* gene p.Tyr1696Asp (Figure 1(g)). This variant has not previously been reported in the UMD-FBN1 mutation database (www.umd.be/FBN1/) and is not present in a large healthy control database (gnomad.broadinstitute.org), but it is predicted to be damaging by UMD-predictor, CADD and MutationTaster2 [12–14].

After genetic testing, a trial with high-dose rGH treatment at a dose 0.06 mg/kg/day was started due to extremely severe dwarfism which resulted in incremental growth of 1.5 cm per 6 months of treatment (Figure 1(e)) with a subsequent increase of IGF-1 level (318.8 ng/ml, between the 50th and 90th percentile) after 3 months of treatment and 151 ng/ml (between the 10th and 50th percentile) after 6 months of treatment accordingly.

**Figure 2:**
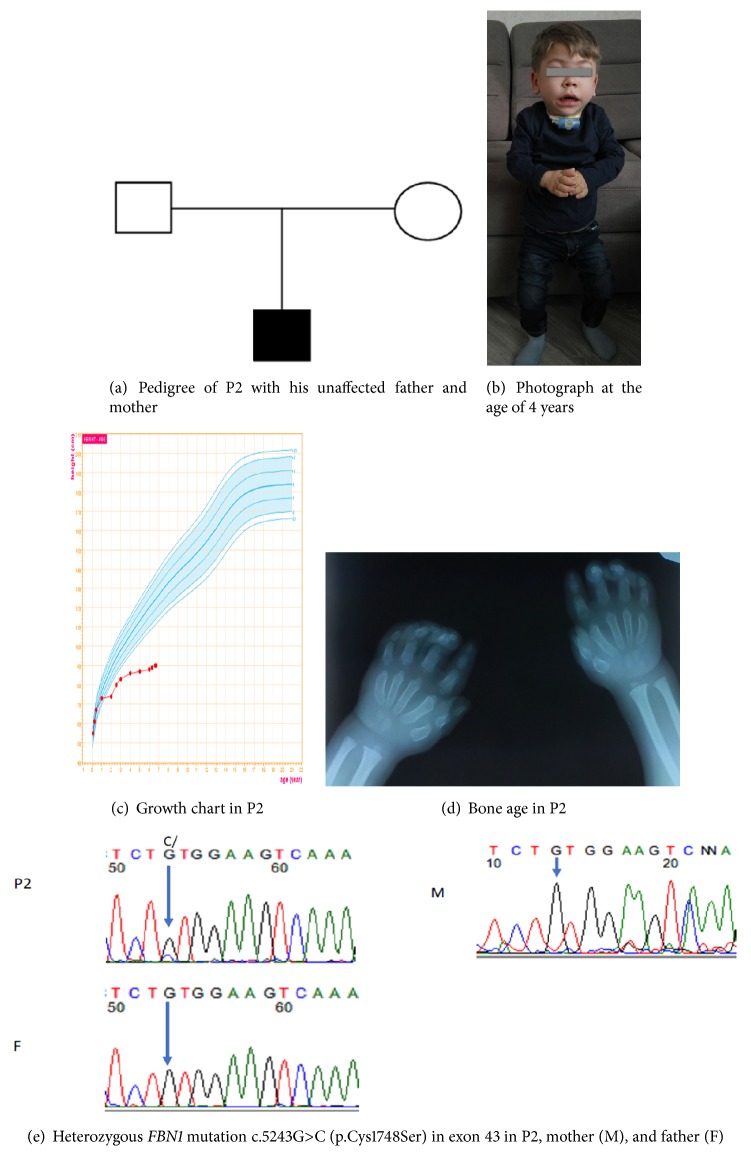


Patient 2 (P2) is a Ukrainian boy who was born as the first child of nonconsanguineous white parents via spontaneous term delivery. He was born at a gestational age of 40 weeks with a birth weight of 4050 g (85^th^-97^th^ percentile) and birth length of 55 сm (>97^th^ percentile). Familial stature was quite tall with a maternal height of 180 cm (+2.9 SD) and paternal height of 195 cm (+3 SD) (Figure 2(a)). Progressive postnatal growth retardation developed beginning at 1 year of age. Prior to 2 years of age, the child had frequent respiratory infections. At the age of 2 years, a tracheostomy was placed due to acute edema of the throat, asphyxia, and pneumonia. In Ukraine, all further attempts to remove the tracheostomy with dilation and resection of pathologic tissue were unsuccessful.

The patient had many dysmorphic features and other physical abnormalities including an indented nasal bridge, elongation of the eye lashes, prominent upper jaw, peripheral edema, thick lips, tapered fingers, stiff interphalangeal joints, and short hands and fingers. At 2 years of age, comprehensive screening for metabolic disorders was completed including mannosidosis, fucosidosis, metachromatic leukodystrophy, Sandhoff disease, lysosomal storage diseases, GM1 gangliosidosis, Krabbe disease, and mucopolysaccharidosis (types 1-3 and 6). However, all metabolic testing was within normal limits. Thereafter, the child was referred to two European clinics in Germany and Denmark with the aim of removing the tracheostomy, but in both cases video laryngoscopy showed large adenoid-like tissue within the rhinopharynx and oropharynx which could cause persistent obstruction. Therefore, it was recommended that the tracheostomy remain in place permanently. Due to the suspicion of mucopolysaccharidosis or mucolipidosis, a targeted next generation sequencing panel of 99 genes was performed; however no variants were identified.

The patient was first examined by an endocrinologist at 4 years of age (Figure 2(b)). Blood samples showed normal hematology, chemistry, and thyroid hormone function. IGF-1 was 41.8 ng/ml (<5th percentile); however clonidine-stimulated GH peak was normal (12.8 ng/ml). IGF-1 generation test showed a good response to stimulation (after three days of GH administration at a dose of 0.03 mg/kg/d IGF-1 was 205.5 ng/ml). However, treatment with rGH at a dose 0.03 mg/kg/day for 5 months did not result in catch-up growth (0 cm per 5 months of treatment) (Figure 2(c)). He had a normal male karyotype (46 XY). His bone age was 2 years (assessed by the Greulich and Pyle method) with noticeable cone-shaped epiphyses (Figure 2(d)). Echocardiography was normal.

Similar to the first patient, comprehensive genetic testing was done at 6 years of age. Chromosomal microarray was negative but whole exome sequencing identified a* de novo* heterozygous mutation in* FBN1* p.Cys1748Ser (Figure 2(e)). Similar to the variant found in the first patient, this mutation was not previously described in the UMD-FBN1 database and was not present in the gnomAD healthy control database, but it is predicted as pathogenic by UMD-predictor, CADD and MutationTaster2 [12–14].

After genetic testing, a trial with high-dose rGH treatment at a dose of 0.06 mg/kg/day was started and showed no improvement in growth (0 cm per 3 months of treatment, Figure 2(c)).

Treatment with anti-inflammatory drugs for his airway issues was started including azithromycin and inhaled or nebulized mometasone.

## 3. Discussion

The Ukrainian Pediatric Growth Hormone Registry was created in 2012 to include children diagnosed with short stature identified by regional Ukrainian pediatric endocrinologists. The number of cases with hypopituitarism in Ukraine in 2016 was 962 (a prevalence of 1 in 7914 for the pediatric population in 2016), and there were an additional 315 cases of Turner syndrome (1:24171). The registry includes also additional patients with syndromic dwarfism who are not receiving GH treatment. However due to the lack of access to genetic diagnostics in Ukraine, a case of GD has not been previously described.

The diagnosis of GD can be based on clinical findings including proportionate short stature, very short hands and feet, progressive joint limitation and contractures, distinctive facial features (round, full face; small nose with anteverted nostrils; broad nasal bridge; thin upper lip with flat philtrum), thickened skin, and progressive cardiac valvular disease [1–4]. Additional features include recurrent respiratory and middle-ear infections, tracheal stenosis, and hepatomegaly [1, 6].

In this report, we describe two patients with molecular defects in* FBN1* leading to severe short stature which was resistant to GH treatment. Both patients remained undiagnosed for many years until exome sequencing confirmed the etiology of their clinical presentations. Both children had facial dysmorphisms (broad nasal bridge, a bulbous nose, elongation of the eye lashes, small hands and fingers, progressive joint limitation, and contractures). However one of the patients (P1) had progressive cardiac pathology which was found at the age of 7 years, and the second patient had severe pathology of the respiratory system with tracheal stenosis requiring tracheostomy placement at 2 years of age. In patients with GD, rapid progression of cardiac pathology has been also described [15, 16] leading to the necessity of timely and adequate cardiac health supervision including the use of valve replacement when indicated. Severe respiratory problems were described as a leading cause of early death in patients with GD [6, 7] and often require tracheostomy placement.

Radiographic findings usually include delayed bone age, broad proximal phalanges, cone-shaped epiphyses, ovoid vertebral bodies, shortened tubular bones of the hands and feet, and small capital femoral epiphyses [1, 4, 17]. Skeletal survey in both our patients showed cone-shaped epiphyses and shortening of tubular bones. One of the patients (P1) had dysplasia of the hip and Madelung deformity with carpal tunnel syndrome requiring a carpal tunnel release. Neither patient had hepatomegaly which was confirmed by abdominal ultrasound.

Genetic testing confirmed two newly identified mutations in* FBN1* (p.Tyr1696Asp and p.Cys1748Ser) which are located in the TB5 domain. Other mutations in this* FBN1* domain are linked with Marfan syndrome and WMS. Sanger sequencing confirmed the presence of the* de novo* variants in each of the patients. The variants were absent in the parents. Although the two reported mutations are new, a few mutations have been described in the same amino acids: p.Tyr1696Cys (X2) [6], p.Cys1748Phe [18], and p.Cys1748Arg [19]. In comparing the phenotypes of patients with the similar variants, it is noteworthy that a patient with p.Tyr1696Cys also required tracheostomy placement at 3 years of age. That patient additionally had mitral stenosis and insufficiency and died at 9 years of age [6]. The patients with p.Cys1748Phe and p.Cys1748Arg had WMS phenotype with ectopia lentis and in the case of the Cys1748Arg mutation an acute thoracic aortic dissection at 38 years of age [18, 19].

There is insufficient data regarding GH treatment in patients with various skeletal dysplasias [20] especially in those with acromelic dysplasia syndromes. However, there have been a variety of reports with conflicting results [21, 22] presumably due to the underlying genetic heterogeneity and different treatment regimens. A trial of recombinant IGF-1 therapy (mecasermin) in a single patient was also previously described [4]. As GD patients are characterized by severe short stature (>3 SD) [6], long-term follow-up is needed to study the advisability of GH treatment regimens in an effort to improve their final height.

Taking into account the severity of dwarfism in our patients (> -6 and -8 SD) with resistance to rGH treatment and severe multiorgan involvement (rapid progression of cardiac pathology and tracheostomy placement), there is a need for further research into alternate treatment modalities. Annual multidisciplinary examination is recommended to provide a comprehensive evaluation of all involved organ systems in order to assess for known comorbidities of GD.

## Abbreviations

AD: Acromicric dysplasia
GD: Geleophysic dysplasia
GH: Growth hormone
rGH: Recombinant GH
WMS: Weill-Marchesani syndrome.
